# A Novel Monitoring Method of Wind-Induced Vibration and Stability of Long-Span Bridges Based on Permanent Scatterer Interferometric Synthetic Aperture Radar Technology

**DOI:** 10.3390/s25113316

**Published:** 2025-05-24

**Authors:** Jiayue Ma, Xiaojun Xue, Guoliang Zhi, Haoyang Zheng, Hanqing Zhu

**Affiliations:** 1 Electric Power and Architecture College, Shanxi University, Taiyuan 030031, China; mjy200408172022@163.com; 2 School of Civil Engineering, Southeast University, Nanjing 210096, China; haoyangzheng@seu.edu.cn (H.Z.); hanqing.zhu@seu.edu.cn (H.Z.)

**Keywords:** PS-InSAR technology, long-span bridge, wind-induced vibration, bridge stability, monitoring method

## Abstract

Long-span structures are highly vulnerable to wind-induced vibrations, which can pose a significant threat to their structural stability and safety. This paper introduces a novel monitoring method that combines Permanent Scatterer Interferometric Synthetic Aperture Radar (PS-InSAR) technology with Auto-Regressive Moving Average (ARMA) models, providing an innovative approach to monitoring wind-induced vibrations in large-span bridges. While previous studies have focused on individual techniques, this integrated approach is largely unexplored and offers a new perspective for structural health monitoring. By collating a series of SAR images and examining phase alterations on the bridge surface, a three-tiered detection methodology is employed to identify stable points accurately. The surface deformation data are then analyzed alongside wind speed and weather data to construct a comprehensive model elucidating the relationship between the bridge and vibrations. The ARMA model is used for real-time monitoring and assessment. Experimental results demonstrate that this method offers precise, real-time monitoring of wind-resistant stability. By leveraging the spatial accuracy and long-term monitoring capability of PS-InSAR, along with the time-series forecasting strength of ARMA models, the method enables data-driven analysis of bridge vibrations. It also provides comprehensive coverage under various conditions, enhancing the safety of long-span bridges through advanced predictive analytics.

## 1. Introduction

Sea-crossing bridges are vital components of the transportation network, playing a key role in improving marine transport infrastructure and holding significant strategic value [1,2]. However, their large spans and high flexibility make them particularly vulnerable to wind loads, which can compromise their safety. Therefore, regardless of the bridge type, it is crucial to ensure stability and optimal structural safety throughout both the construction and operational phases [3]. This is especially important for long-span bridges [4], including cable-supported structures, due to their distinct structural characteristics and sensitivity to dynamic loads [5].

Bridge wind-resistant stability not only encompasses structural safety [6,7] but also considers the structure’s adaptability to the natural environment [8,9,10] and human factors [11,12]. It refers to the ability of the bridge to maintain stability and prevent failure when subjected to wind loads. Achieving a comprehensive analysis of bridge stability requires addressing the intricate interactions among structural resilience, environmental impacts, and human usage patterns. This holistic approach ensures that long-span bridges can endure dynamic loads and environmental challenges while preserving their integrity over time. Additionally, it enhances the reliability and longevity of bridges, ensuring they meet both environmental and societal needs.

Given the characteristics of large spans and flexible structures, long-span bridges are particularly vulnerable to wind loads. Wind-induced vibrations can cause structural instability and fatigue damage, thereby reducing the overall service life of the bridge [13]. These vibrations can also lead to resonance phenomena, which can exacerbate structural stresses and accelerate material degradation. Therefore, monitoring and analyzing these vibrations is essential for maintaining the stability and longevity of long-span bridges, given their unique structural configuration and shape [14,15]. Advanced monitoring techniques, such as Permanent Scatterer Interferometric Synthetic Aperture Radar (PS-InSAR) technology, provide high-precision, real-time data that can help identify potential issues before they become critical. By integrating these technologies with traditional stability monitoring methods, engineers can develop more effective maintenance and mitigation strategies, ultimately enhancing the resilience and reliability of long-span bridges in the face of dynamic wind loads.

Among the various studies reported in recent years, PS-InSAR technology, which can detect differential displacements at specific surface points, has greatly advanced the long-term monitoring of surface deformations in urban and infrastructure environments [16,17]. Specifically, it utilizes radar satellites (e.g., COSMO-SkyMed, Sentinel-1A, and TanDEM-X) to observe the Earth and obtain high-precision, high-resolution ground deformation data. This innovative approach is particularly beneficial for monitoring long-span bridges [18]. By employing PS-InSAR technology, deformation data can be acquired under various environmental conditions, including those caused by dynamic loads such as wind-induced vibrations. These vibrations can significantly impact the structural integrity and safety of bridges [19,20]. Analyzing these data allows for the calculation of the frequency and amplitude of vibrations across different wind speeds and directions, facilitating an accurate evaluation of wind-resistant stability. For long-span bridges, PS-InSAR technology overcomes the limitations of traditional sensor-based monitoring methods by providing relatively precise and detailed surface deformation data, enhancing spatial coverage, data continuity, and adaptability to environmental changes. Additionally, PS-InSAR technology enables continuous, all-weather monitoring, promptly identifying potential risks and providing robust data support for the design of bridge stability and safety [21]. These investigations not only help engineers gain a deeper understanding of wind-induced vibration characteristics but also offer valuable guidance for enhancing the wind-resistant design of bridges [22].

When this novel technology is applied to large-scale monitoring of vibration-sensitive bridge groups in regions comprising multiple large-span bridges, it also shows excellent potential. Recently, advances in cloud computing and parallel processing have significantly improved computational efficiency. These technologies allow for the simultaneous monitoring of multiple large-span bridges, providing a comprehensive view of the overall structural health of the bridge groups [23]. Regarding satellite revisit rates, while PS-InSAR relies on the availability of frequent satellite passes, the method in our study is adaptable to the revisit cycle of current satellite missions. The high-precision data collected can be used to detect early signs of wear and tear, structural weaknesses, and other potential issues across the entire bridge group [24]. This technology facilitates efficient resource allocation for maintenance and repair by identifying the most critical areas that require immediate attention. Although the initial setup for PS-InSAR monitoring can be expensive due to satellite data acquisition, its long-term costs are generally lower than those of traditional continuous sensor-based monitoring methods. Moreover, PS-InSAR data can be integrated using standard data formats and customized interfaces for real-time visualization. Its integration with other advanced monitoring systems may also support the development of a smart infrastructure management platform, further optimizing large-scale bridge maintenance and operations.

Extensive research and applications regarding bridge stability monitoring have been conducted over the past few decades [25,26,27,28]. Fenerci et al. [29] collected long-term wind-induced vibration data from a bridge and used it to establish a baseline model, offering a precise depiction of the bridge’s long-term performance changes. This model enhanced the reliability and accuracy of the monitoring data but required substantial data preprocessing and analysis. Wedel and Marx [30] employed a machine learning algorithm for bridge health monitoring, processing extensive data and automatically identifying patterns through sensors that collect structural and environmental information, including vibration, deformation, temperature, and humidity. However, the algorithm’s accuracy and reliability depended on the quality and quantity of available data, requiring extensive training with high-quality datasets, which are often limited in bridge monitoring practices. Mufti et al. [31] presented numerous cases on bridge health monitoring, providing real-time data on bridge responses, including strain, vibration, and temperature. This aids in identifying potential damage, abnormalities, and predicting the bridge’s remaining lifespan. Additionally, it offers insights into the dynamic response of bridges under various loads and environmental conditions, enhancing stability and safety evaluations. However, practical application requires numerous sensors and equipment, increasing installation and maintenance costs.

The aforementioned studies highlight the progress and challenges in bridge stability monitoring, emphasizing the need for advanced methods to overcome existing limitations and ensure accurate, reliable, and cost-effective monitoring. In response to these shortcomings, a novel approach utilizing PS-InSAR technology has been proposed to monitor wind-induced vibration and wind-resistant stability in long-span bridges. This method employs radar interferometry to observe bridge stability under wind loads. By integrating PS-InSAR technology with vibration monitoring techniques and wind engineering theories, it enables real-time, high-precision monitoring of wind-induced vibrations in long-span bridges under all weather conditions. Specifically, the proposed method not only leverages the spatial accuracy and long-term monitoring capability of PS-InSAR but also incorporates the time-series forecasting strength of Auto-Regressive Moving Average (ARMA) models to achieve data-driven analysis of bridge vibrations. This integrated approach has not been extensively explored and offers a new perspective for structural health monitoring of long-span bridges, ensuring their safety, stability, and functionality. It enhances our understanding of bridge stability monitoring and offers valuable insights for designing wind-resistant stability in long-span bridges.

## 2. Methodology for Monitoring Wind-Induced Vibration and Stability

### 2.1. Time Series Data Acquisition

The selection of the Sentinel-1 Synthetic Aperture Radar (SAR) image as the primary data source for monitoring wind-induced vibration and stability in long-span bridges is based on its advanced capabilities. The Sentinel-1 satellite system is equipped with a C-band synthetic aperture radar sensor, which enables the observation of targets under all-day and all-weather conditions. The system offers four distinct operational modes, namely, wide interference mode, strip mode, band mode, and extremely wide mode. To monitor wind-induced vibration and stability in long-span bridges, SAR image information is collected in a systematic manner using the wide interference mode.

By employing PS-InSAR technology, phase change information from monitoring points on the bridge surface is extracted, resulting in the generation of time series data pertaining to the deformation of the bridge surface. The dataset comprises a series of SAR images of the bridge surface, which are divided into two categories: main images and auxiliary images. Subsequently, the images are registered to create a differential interference map [32], which provides insights into the bridge’s deformation from multiple perspectives. An external digital elevation model is employed to eliminate terrain phase information from the SAR images, thereby facilitating the identification of candidate permanent scatterer (PS) points. PS points are pixel points with high phase stability in the time series of SAR images, representing various stable ground objects [33]. Once the candidate PS points have been identified, the final PS points are determined based on their phase stability.

It is imperative to capture a multitude of SAR images of extensive bridge surfaces to identify pixels that exhibit high stability and scattering characteristics, which are defined as permanent scatterer (PS) points. In general, PS points in SAR images are indicative of hard targets, such as corners and boundaries [34]. To optimize the precision and computational efficacy of PS-InSAR technology, it is recommended that a greater number of PS points be selected. It is imperative that these points demonstrate high reliability to avoid potential inaccuracies in monitoring the stability of long-span bridges due to inappropriate PS point selection.

The crucial element in determining PS points is the identification of an appropriate characteristic function and selection principle. The single-threshold detection method, when employed to select PS points, carries the risk of either prioritizing strong scattering characteristics in SAR images while overlooking the stability of PS points, or vice versa. This can result in erroneous decisions being made [35]. To guarantee the most precise extraction of PS points, a three-stage detection method is employed. This method considers the amplitude information, average coherence coefficient, and amplitude deviation index of the PS points, thereby addressing the high signal-to-noise ratio characteristics of PS echo signals in long-span bridge SAR images [36]. A single-threshold parameter often faces limitations in areas with natural surface coverage and complex environmental conditions. Therefore, two threshold parameters, the amplitude threshold and the average coherence coefficient, are established to identify potential PS points in the SAR images. This process was well validated in similar studies [37,38] and showed improved monitoring performance in practical engineering applications [39]. Subsequently, the amplitude deviation threshold method, which considers the stability of PS points, is employed to determine the final PS points for monitoring the wind-resistant stability of bridges.

The three-stage method for detecting PS points in long-span bridge SAR images encompasses the following key processes:

(1) Computation of Coherence Coefficient

In the context of the time series of long-span bridge SAR images, the expression for calculating the coherence coefficient for each pixel in the image is as follows:

(1) $$\gamma =\frac{\left|\sum_{i=1}^{m}\sum_{j=1}^{n}M\left(i,j\right)S^{*}\left(i,j\right)\right|}{\sqrt{\sum_{i=1}^{m}\sum_{j=1}^{n}{\left|M\left(i,j\right)\right|}^{2}\sum_{i=1}^{m}\sum_{j=1}^{n}{\left|S\left(i,j\right)\right|}^{2}}}$$

where *S* (*i*, *j*) and *M* (*i*, *j*) represent the pixels and interferograms of SAR images, respectively. And * denotes the complex conjugate operation.

The SAR image dataset consists of *N* + 1 images, generating *N* interferograms. The coherence coefficient sequence for each pixel in the image is $\gamma_{m}$.

(2) Based on the time series of SAR images, the average expression for calculating the pixel coherence coefficient in SAR images is as follows:
  (2) $$\bar{\gamma }=\sum_{i=1}^{N}\frac{\gamma_{i}}{N}$$

(3) To filter out water and vegetation in the long-span bridge SAR image, a coherence coefficient threshold ($\gamma_{A}$) is set, which is influenced by factors such as the image resolution and surface characteristics.

(4) Compute the amplitude values of the time series ($m_{i}$).

(5) Calculate the amplitude threshold ($T_{A}$), which is given by:
  (3) $$T_{A}=\min\left\{\frac{1}{i}\sum_{i=1}m_{i}\right\}$$

(6) The process allows for the determination of PS points in SAR images of long-span bridges. A pixel is classified as a candidate PS point if it simultaneously meets the threshold criteria for the correlation coefficient and satisfies the aforementioned criteria. Otherwise, the pixel is designated as a non-PS point.

(7) Calculate the standard deviation ($\sigma_{i}$) of the time series amplitude in long-span bridge SAR images.

(8) Compute the amplitude deviation index ($D_{A}$) of the candidate PS points using the following expression:
  (4) $$D_{A}=\sigma_{i}/m_{i}$$

It quantifies the variation in amplitude stability over time, assessing the reliability of the PS points and identifying those with significant amplitude fluctuations.

(9) PS point determination

Establish the stability threshold ($T_{d}$), when $D_{A}\leq T_{d}$ classifying the candidate PS point as a PS point; otherwise, designate the pixel as a non-PS point.

In the interference map of the surface SAR image for the *i*th long-span bridge, the expression for the phase residual of the *x*th PS point is as follows:

(5) $$\varphi_{x,i}=\varphi_{def}+\varphi_{a}+\varphi_{orb}+\varphi_{\varepsilon }+n$$

where $\varphi_{def}$ and $\varphi_{x,i}$ denote the deformation phase in the radar line-of-sight direction and the phase value of pixel points, respectively. $\varphi_{orb}$ and $\varphi_{a}$ represent the phase of the orbit error and the delay phase difference of the transit atmosphere, while *n* and $\varphi_{\varepsilon }$ stand for noise phase and digital elevation model (DEM) error phase, respectively.

In a specified distance range, $\varphi_{a}$, $\varphi_{def}$ and $\varphi_{orb}$ exhibit spatial correlation, while $\varphi_{\varepsilon }$ and *n* are spatially uncorrelated, averaging to 0. Considering the pixels of the long-span bridge SAR image denoted by *x*, with the center of the circle and a fixed length as the radius, the expression for the average value of all phases in this circular region is as follows:

(6) $${\bar{\varphi }}_{x,i}={\bar{\varphi }}_{def}+{\bar{\varphi }}_{a}+{\bar{\varphi }}_{orb}$$

Combining Equations (5) and (6), the following expression can be obtained:

(7) $$\varphi_{x,i}-{\bar{\varphi }}_{x,i}=BK-\left({\bar{\varphi }}_{def}-\varphi_{def}\right)+\left({\bar{\varphi }}_{a}-\varphi_{a}\right)+\left({\bar{\varphi }}_{orb}-\varphi_{orb}\right)$$

where *B* and *K* represent the vertical baseline and the scale factor, respectively.

The least squares method was employed to process the pixels in the long-span bridge SAR images, resulting in the estimation of the proportional coefficient [40]. The temporal coherence of a pixel in the SAR image *x* is defined by the following expression:

(8) $$\gamma_{x}=\frac{1}{N}\left|\sum_{i=1}^{N}\exp\left(\varphi_{x,i}-{\bar{\varphi }}_{x,i}-{\hat{\varphi }}_{\varepsilon }\right)\right|$$

where *N* represents the number of interferograms, ${\hat{\varphi }}_{\varepsilon }$ denotes the estimated value of $\varphi_{\varepsilon }$. It is noted that temporal coherence serves as an indicator of the phase stability of pixels.

**Figure 1:**
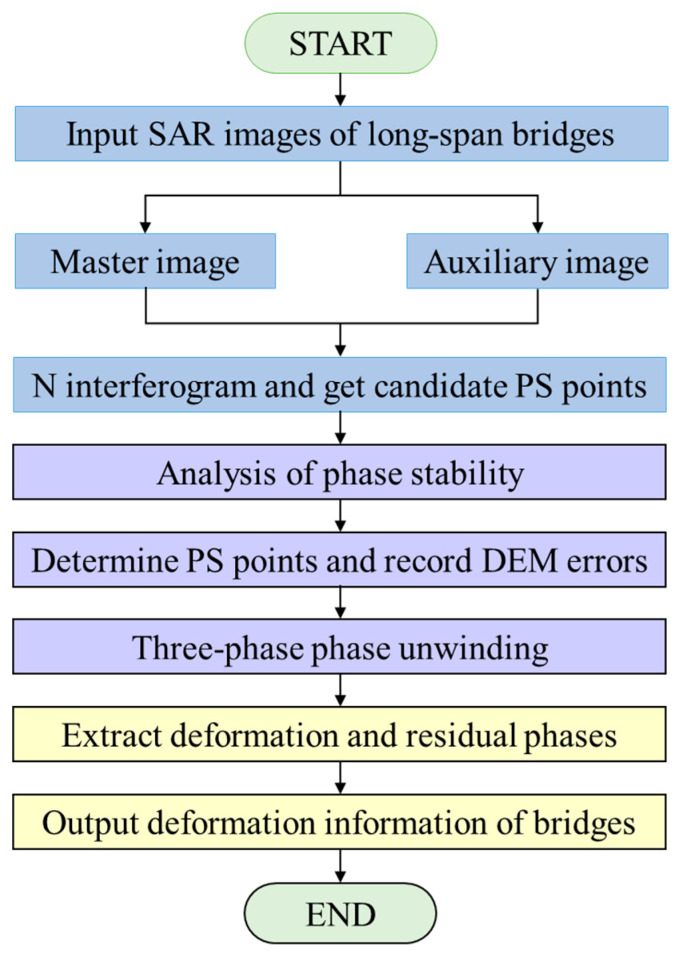
Surface time series data processing flowchart.

The utilization of PS-InSAR technology facilitates the interferometric processing of multiple SAR images of long-span bridges, with a particular emphasis on the PS of the primary image. The detection of PS points in SAR images enables the acquisition of valuable deformation information about long-span bridges. The process of obtaining time series data is illustrated as a flowchart in Figure 1, with differently colored boxes to distinguish the various phases.

The flowchart illustrates the process of applying PS-InSAR technology to obtain time series data. This involves dividing the collected SAR images of long-span bridge surfaces into main and auxiliary images. After this division, a three-stage PS point detection method is used to identify the PS points. The phase error of each PS point is then calculated, followed by the extraction of deformation and residual phases using a three-dimensional phase unwrapping method. This process ultimately determines the final deformation information for the long-span bridge surface.

### 2.2. Modeling the Relationship Between Bridge Structure and Vibration

Considering wind speed and meteorological data, a comprehensive model is developed to describe the relationship between long-span bridges and wind-induced vibrations. The interaction between wind and the bridge is complex, involving multiple factors such as structural shape, natural wind direction, wind load, and the dynamic characteristics of the bridge. The wind field’s motion direction, spatial distribution, and velocity are significantly influenced by turbulence in the near-surface boundary layer, which exhibits noticeable randomness and instability. When fluctuating wind encounters the non-streamlined cross-section of a bridge, it produces flow around the bluff body, resulting in a highly complex wind field distribution. These dynamic effects cause the bridge to vibrate, which in turn affects the airflow. The structural vibrations alter the airflow forces, creating a complex interaction between the wind and the bridge [41,42]. Considering both the average and fluctuating wind forces and wind pressure distributions over the long-span bridges, wind loads primarily act on the tower and main girder. Wind loads on long-span bridges mostly consist of static wind loads and dynamic wind loads. The average wind provides the static wind load, which is described by the following equations:

(9) $$F_{res}=\frac{1}{2}\rho V^{2}hLC_{D}$$

(10) $$F_{lift}=\frac{1}{2}\rho V^{2}hLC_{L}$$

where $F_{res}$ and $F_{lift}$ are the drag and lift forces, respectively; ρ and *V* represent the air density and the wind speed, respectively; *h* and *L* denote the height and length of the bridge subjected to the average wind, respectively; and $C_{D}$ and $C_{L}$ are the drag and lift coefficients under the static wind load, respectively.

When a long-span bridge is exposed to a wind field, it may exhibit an aeroelastic dynamic response, which is most commonly observed under pulsating winds or turbulent conditions. While such a phenomenon can also occur under laminar flow, it generally requires higher wind speeds to induce significant dynamic responses. For simplicity in the initial theoretical framework, laminar flow was assumed, particularly in the early stages of stability analysis, to facilitate a more manageable set of equations for basic analytical purposes. In this context, the long-span bridge is subjected to dynamic wind load, experiencing both forced and self-excited vibration. The differential equation for flutter in the self-excited vibration of a long-span bridge can be expressed as follows [42]:

(11) $$P_{ae}(s,t)=m(s)\ddot{X}(s,t)+\gamma (s)\dot{X}(s,t)+g(s)X(s,t)$$

where $m\left(s\right)$ and $\gamma \left(s\right)$ are the mass and the damping of bridge members, respectively; $X\left(s,t\right)$ and $g\left(s\right)$ are the position vector and member stiffness of the structure nodes, respectively. Moreover, $P_{ae}(s,t)$ is the aerodynamic force, typically including both the drag force $F_{res}$ and the lift force $F_{lift}$, with the specific form depending on the bridge’s geometry and wind conditions [42].

The process of establishing the differential equation for wind load and bridge flutter, as described above, quantifies the interaction between the long-span bridge and wind-induced vibration, thereby establishing a relationship model between them. In practice, the turbulent flow more accurately represents real-world conditions, and buffeting effects must be considered for a more precise stability analysis [5]. Furthermore, it is possible to study the homogeneous equation to determine the eigenvalues.

### 2.3. Wind-Induced Vibration and Stability Monitoring

The acquired time series data from the long-span bridge surface, combined with the established relationship model of wind-induced vibrations, provide the basis for real-time monitoring. Based on this, bridge vibrations can be effectively assessed through the automatic search order determination method. This approach enables the timely identification of anomalous vibration occurrences in long-span bridges.

In this time series analysis, the ARMA model, which combines the autoregressive and moving average components, is employed. The autoregressive component denotes the interconnection between the present and preceding values within the series, whereas the moving average component signifies the relationship between the current value and the random error term. This statistical model is an effective means of characterizing the randomness, trends, and periodicity present in the data. $\left\{a_{t},t\in Z\right\}$ is utilized to denote the stationary sequence, where the mean and variance of the average sequence are independent. The value of $\sigma_{a}^{2}$ must satisfy $E\left(a_{t}\right)=0$ and $E\left(a_{t},a_{t-k}\right)=\left\{\begin{array}{c} \sigma_{a}^{2},k=0\\ 0,k\neq 0 \end{array}\right.$.

In the context of bridge vibrations, the structural responses are often of large amplitude and may exhibit nonlinear or transient features. To address these issues and meet the assumptions of the ARMA model, a detrending process was applied to the deformation time series before modeling. This process involved removing long-term trends and low-frequency components using polynomial fitting and differencing, which improved the stationarity of the data. After preprocessing, the observed sequence of wind-induced vibration in long-span bridges ($\left\{x_{t},t\in Z\right\}$) can be approximated as a stationary time series and is assumed to satisfy the following linear difference equation:

(12) $$x_{t}-\phi_{1}x_{t-1}-\phi_{2}x_{t-2}-\cdots -\phi_{p}x_{t-p}=a_{t}-\theta_{1}a_{t-1}-\theta_{2}a_{t-2}-\cdots -\theta_{q}a_{t-q}$$

Equation (12) is an autoregressive sliding average model of $X\left(t\right)$ for the wind-induced vibration time series, denoted by $ARMA\left(p,q\right)$, where $\phi_{i}$ and $\theta_{i}$ are the autoregressive and moving average parameters, respectively. $\left\{a_{t}\right\}$ is the white noise sequence of the regression equation.

When $\phi_{i}=0$ or $\theta_{i}=0$, Equation (12) can be transformed as follows:

(13) $$x_{t}=\phi_{1}x_{t-1}+\phi_{2}x_{t-2}+\cdots +\phi_{p}x_{t-p}+a_{t}$$

(14) $$x_{t}=a_{t}-\theta_{1}a_{t-1}-\theta_{2}a_{t-2}-\cdots -\theta_{q}a_{t-q}$$

Equations (13) and (14) are the *p*-order autoregression model and the *q*-order sliding average model of $X\left(t\right)$ for the time series.

The model $ARMA\left(p,q\right)$ is a highly representative model in timing analysis. Unlike traditional regression models, it adopts random variables associated with the actual sequence at time *t* of the bridge vibration. This model enhances the continuity and memory of past deformation data and integrates the deformation criteria, thereby improving the monitoring of wind-induced vibration and stability.

**Figure 2:**
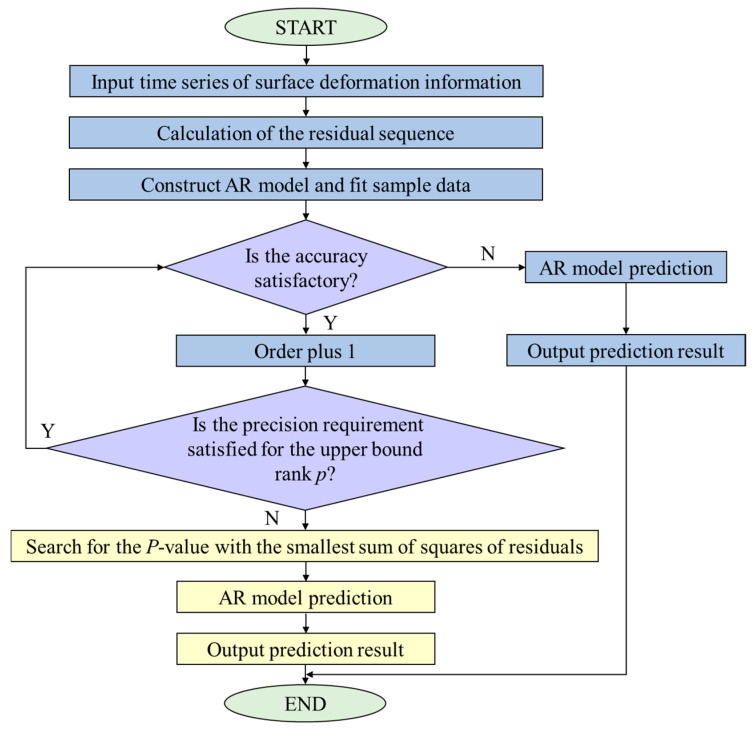
Flowchart of the automatic order determination method.

The determination of the order of the ARMA model is of paramount importance for the monitoring of vibrations and stability. The present study employs the automatic search and order determination method to ascertain the model order, as illustrated in the flowchart in Figure 2. The boxes are colored differently to distinguish the various stages of the process, which helps enhance the clarity of the flowchart.

It depicts the order determination of the ARMA model, which is completed by searching for the order that corresponds to the minimum residual sum of squares. This approach is applicable when the relative error in fitting wind-induced vibration data is below 5%, or when the upper bound of the *p*-order model fails to meet the requisite accuracy standards.

In consideration of the fundamental structure of the long-span bridge, the critical wind speed for the influence of wind-induced vibration on stability is expressed as follows [42]:

(15) $$V_{cr}=\kappa f_{t}\omega$$

where κ and $f_{t}$ represent structural critical parameters and the symmetric torsional fundamental frequency of the bridge structure, respectively, with units of m⋅s and Hz; ω denotes the wind-induced vibration frequency, with a unit of Hz.

According to the ARMA model output for wind-induced vibration in long-span bridges, the wind-induced response is categorized into different grades (see Table 1 for details). This classification provides a refined understanding of the varying effects of wind on the bridge vibration, facilitating the assessment of wind-induced vibrations in bridges.

The relationship model between the long-span bridge and wind-induced vibration uses time series data from surface deformation monitoring points as input for the ARMA model. The output consists of monitoring results for wind-induced vibrations in the bridge. By comparing these results with the various grades of wind-induced response, the response level of the long-span bridge can be determined. This real-time monitoring and assessment approach enables the timely detection of anomalous vibration phenomena in long-span bridges.

## 3. Example Analysis and Practical Implementation

A cable-stayed river-crossing bridge, with a span of 1700 m and a deck width of 27 m, has been selected for the investigation of wind-induced vibration and stability monitoring. As the longest bridge in Wuhan, Hubei Province, China, it features six lanes in both directions and a total length of 30 m. When applying the aforementioned novel monitoring method to this example bridge, environmental parameters will have a significant impact on the results, with varying environmental influences potentially leading to distinct outcomes. The environmental parameters mainly include wind speed, wind attack angle, and gust frequency and intensity. Wind speed affects the magnitude of aerodynamic loads, while the wind attack angle influences load distribution and localized deformation patterns. Gust frequency and intensity contribute to dynamic loading, potentially inducing short-term peaks or increased displacement variability. As noted in Section 2.2, these parameters serve as external excitations in the monitoring of wind-induced vibration and stability. In this study, the environmental parameters are considered to include both static and dynamic wind, based on long-term statistical data from actual meteorological observations near the bridge site, as detailed in Table 2. It should be noted that the wind attack angle refers to the instantaneous angle between the wind direction and the bridge axis, while the average wind attack angle represents its statistical mean over the observation period.

Furthermore, to obtain SAR image data for the example bridge, Sentinel-1A ascending orbital images were collected daily from 10 October to 31 October 2020. To minimize spatiotemporal decorrelation, interference processing was performed only between adjacent images to preserve the original spatial resolution. The SRTM3 digital elevation model was used to remove topographic phases, and precise orbit ephemerides were applied to correct orbital errors. The optimal main image was then selected from the SAR image dataset for long-span bridges. The temporal and spatial baseline distribution of these images is presented in Table 3, which includes 10 sets of SAR image sequences. The maximum temporal baseline is 3.5 d, and the maximum vertical spatial baseline is 725 m. When used for vibration monitoring of long-span bridges, attention should also be given to the Doppler centroid frequency difference, as both are crucial for monitoring accuracy. Moreover, the impact of inaccuracies in the north–south direction should also be considered theoretically. Given that the primary deformation in this study occurs along the east–west and vertical directions, this effect is minimal and does not significantly affect the monitoring results.

**Figure 3:**
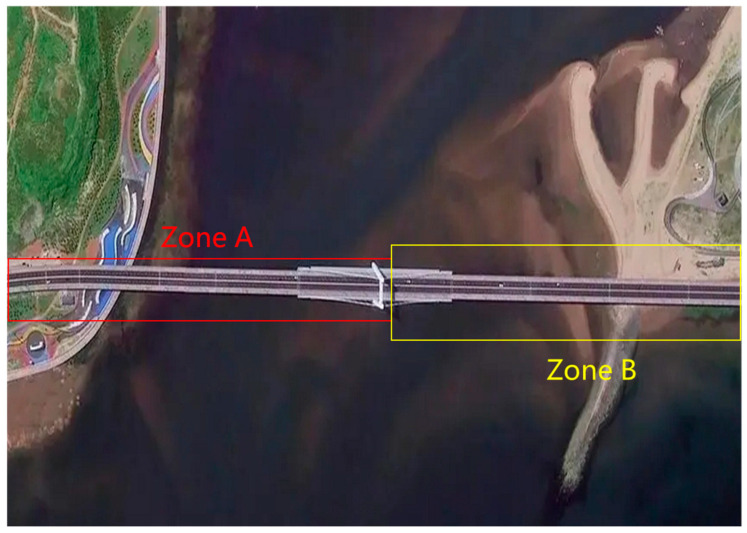
SAR image No. 3 for the long-span bridge.

The results also illustrate the efficacy of this methodology in establishing the temporal and spatial baseline distribution. Notably, the baseline parameters are all zero in the temporal and spatial baseline distribution of SAR image No. 3 for the long-span bridge, indicating minimal distortion and an optimal reference position. Consequently, SAR image No. 3 is selected as the primary image for monitoring wind-induced vibration and stability. The original SAR image No. 3 is depicted in Figure 3 for reference.

**Figure 4:**
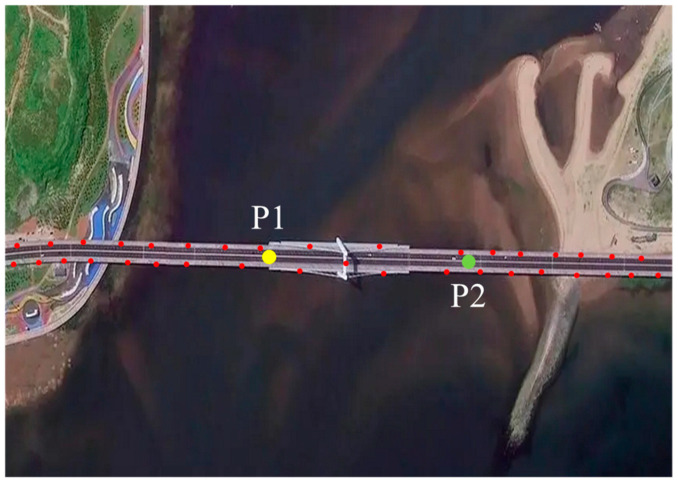
Extraction of PS points.

This image illustrates that the SAR images collected provide an overall view of the long-span bridge, capturing its structural layout and surrounding environment, which are essential for establishing spatial references. These images serve as the basis for monitoring wind-induced vibration and stability. The PS points extracted by the three-level detection method are shown in Figure 4, highlighting the distribution and density of stable points for precise monitoring.

The three-level detection method, which incorporates the amplitude deviation index, amplitude information, and the average coherence coefficient of long-span bridge SAR images, yields effective and reliable PS points. It is worth noting that the distribution of the extracted PS points is concentrated near the bridge, further confirming the effectiveness of this method. Moreover, it is worth noting that the density of PS points should be higher in regions critical for the precise monitoring of wind-induced vibration and stability.

**Figure 5:**
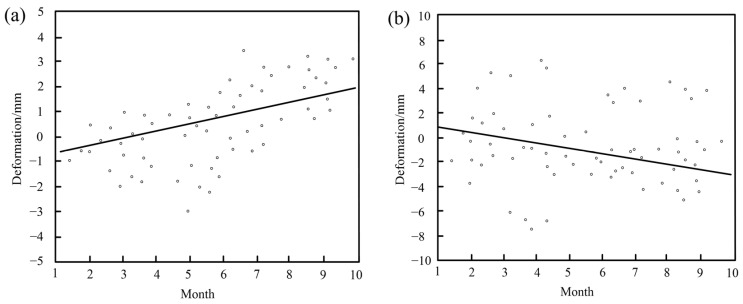
Time series of bridge cumulative deformation during the study period: (**a**) Point P1 in zone A and (**b**) Point P2 in zone B.

To investigate the impact of wind-induced vibration on the cumulative deformation of this long-span bridge, a deformation time series analysis is conducted at key locations on the main bridge. Two characteristic points were selected based on the bridge’s linear features: Point P1 at the interface of the cable-stayed and non-cable-stayed sections, and Point P2 at the center of the span between the tower and the opposite bank. These points are located in zones A and B, as shown in Figure 3 and Figure 4. The monitoring results of cumulative deformation during the study period are presented in Figure 5, showing distinct deformation rate grades and trends. The straight line was obtained by fitting a linear regression model to the deformation data using the least squares method. It reflects the bridge’s deformation behavior over a long monitoring period, approximating the relationship between cumulative deformation and time.

It can be found that the periodic fluctuation of deformation at Point P1 in zone A, within a range of ±4 mm, is influenced by wind conditions. In contrast, Point P2 in zone B demonstrates a stable oscillatory trend with periodic characteristics, exhibiting fluctuations within a range of ±8 mm. These fluctuation ranges align with previous studies [43,44], confirming the reliability of the monitoring results. The slopes of the linear fitting equations for Points P1 and P2 are 0.21 and 0.15, respectively. Although the deformation rates at these points are low, significant fluctuations were observed during the study period. Notably, the large settlement indirectly suggests that some deformations of the bridge may be resilient. Furthermore, the short-term deformation caused by wind-induced vibrations may exhibit nonlinear characteristics, such as sudden displacements and rapid changes in amplitude or phase. These dynamic effects, influenced by multiple factors, are distinct from the long-term trends modeled through linear analysis and are addressed using time-series statistical methods.

**Figure 6:**
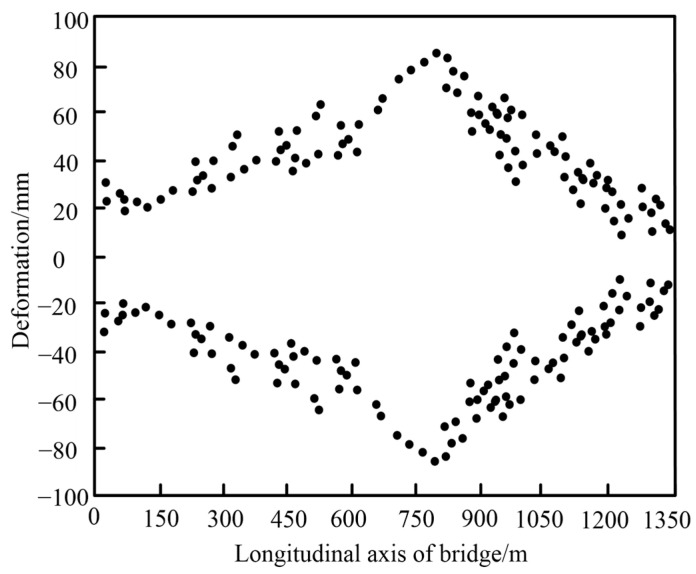
Deformation interval of the PS values.

Figure 6 shows the deformation interval of PS values along the bridge length, derived from analyzing the deformation and displacement of the bridge over the observation period. Through statistical analysis, a deeper understanding of the bridge’s deformation patterns under wind forces is achieved, enabling a more accurate perception of its dynamic structural behavior.

The deformation and displacement of the long-span bridge are symmetrically distributed around the zero axis, reflecting a pattern consistent with expected oscillations under wind forces. Notably, the middle section of the main bridge exhibits the greatest displacement, with fluctuations exceeding those observed at the bridge ends, indicating heightened sensitivity in this central region. The maximum displacement ranges from −100 mm to 100 mm, demonstrating substantial oscillatory movement. Moreover, symmetry along the bridge’s length is evident, with the symmetrical position located approximately 800 m along the longitudinal axis of the bridge. This symmetric distribution implies that displacement from this position toward both ends of the bridge occurs with comparable intensity. This balanced behavior likely reflects the bridge’s structural design, which is intended to accommodate and dissipate wind-induced vibrations effectively.

**Figure 7:**
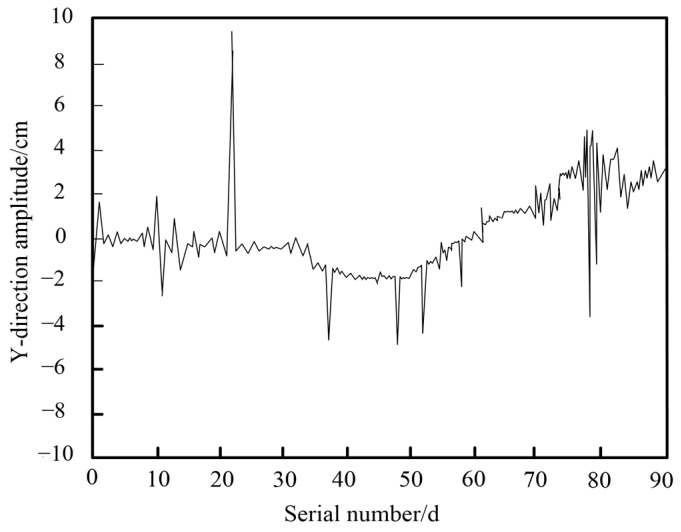
Result of monitoring time series.

To further analyze the wind-induced vibrations, the non-stationary time series of surface deformation information is processed using the previously mentioned method in this paper. Figure 7 presents the statistical results of wind-induced vibration and wind-resistant monitoring time series for this long-span bridge, after removing trend components. These results provide valuable insights into the bridge’s dynamic response under wind loads, enhancing our understanding of its resilience and stability.

**Figure 8:**
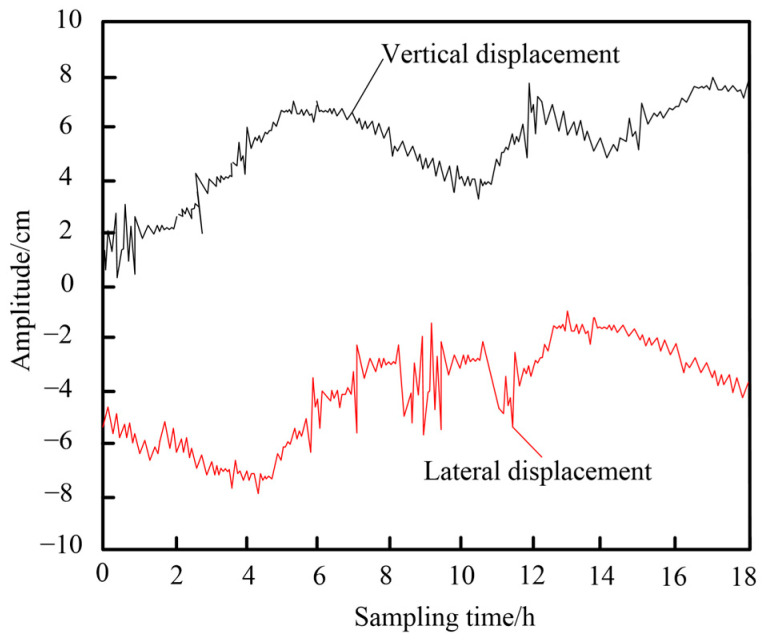
Wind-induced vibration time-history.

It demonstrates the effective processing of wind-induced vibration time series signals for the long-span bridge using the proposed method. By removing the trend component, a smoother residual series curve is obtained, significantly enhancing the monitoring performance for wind-induced vibration and stability. Importantly, the time series monitoring results reveal the largest positive and negative Y-direction displacements near serial numbers 23 and 78, forming sharp peaks. This indicates an increase in instantaneous wind force at these points, causing abrupt changes in the vibration mode of the bridge. This pattern reflects the rapid nonlinear response of the structure to external forces. Additionally, other notable negative Y-direction displacements appear around serial numbers 12, 37, 48, 52, and 59. These patterns support the reliability of the proposed monitoring method for capturing critical fluctuations over extended periods, validating its application in long-term wind-induced vibration analysis. A monitoring point is randomly selected, and the time-history curve of wind-induced vibration at this specific point is shown in Figure 8.

During the 18 h effective sampling period, the vertical displacement of the bridge under wind influence initially increased sharply, then decreased, followed by another increase that stabilized at a certain amplitude with minor fluctuations. This dynamic behavior reflects the variation in wind loads and the bridge’s vibration response to these real-time changes. In contrast, the lateral displacement initially showed small increases, then sharply decreased with larger fluctuations, eventually stabilizing at a moderate amplitude with a gradual downward trend. The difference between vertical and lateral displacement patterns highlights distinct directional sensitivities in the bridge’s structural behavior, with the greater vertical displacement indicating a higher susceptibility of vertical vibration modes to wind-induced forces.

In summary, the results demonstrate the effective acquisition of wind-induced vibration data for monitoring points on long-span bridges. These data represent a crucial indicator for assessing the wind-resistant stability of these structures. By capturing real-time vibration patterns, the data provide insights into how the bridge responds to varying wind conditions, highlighting potential areas of concern that may not be detectable through static analysis alone. The monitoring system’s ability to detect changes in the amplitude, frequency, and phase of the vibrations allows for a comprehensive evaluation of the bridge’s dynamic behavior. The test results detailing the wind-induced vibration and stability of long-span bridges monitored using this method are presented in Table 4.

Specifically, it presents the monitoring data for five randomly selected points on the bridge in both static and dynamic wind environments. The results demonstrate that this method effectively captures the vibration amplitude and frequency of the bridge under both conditions. This capability is critical for assessing how bridges respond to varying wind conditions and understanding their structural behavior under different loading scenarios. A comparison with actual data provided by the Department of Bridge Maintenance and Management (DBMM) reveals minimal discrepancies, validating the high accuracy of this method in wind-resistant stability monitoring. By using parameters such as wind speed, amplitude, and frequency of wind-induced vibrations, this method provides a comprehensive assessment of the long-span bridge’s response to wind forces. It allows for precise tracking of the dynamic behavior of the structure over time, identifying changes that may signal issues with the bridge’s stability. Therefore, this method serves as a reliable tool for wind-induced vibration monitoring, offering a critical basis for evaluating the long-term stability of long-span bridges under varying wind conditions.

The performance of the proposed monitoring method in the case of a cable-stayed bridge has been thoroughly discussed in the previous section. When applied to suspension bridges, which have longer and more flexible spans, the method can also effectively monitor the structural deformations [45]. The main challenge lies in capturing larger amplitude responses at higher frequencies, but with appropriate preprocessing of the data, the method is well-suited for detecting such deformations. In terms of climatic conditions, the method is adaptable to varying factors such as wind speed, temperature, and humidity. In regions with high winds or extreme weather, it can reliably monitor the dynamic response of bridges. However, variations in these conditions may affect the accuracy of long-term deformation trends, necessitating further calibration and validation based on local conditions.

## 4. Conclusions

This study introduces an innovative method for monitoring wind-induced vibrations and stability in long-span bridges by integrating PS-InSAR technology with traditional stability monitoring techniques. The proposed method exhibits high precision and real-time monitoring capabilities, markedly enhancing the detection and assessment of structural deformations. Through a case of bridge temporal deformation analysis, the following conclusions are obtained:

(1) The longitudinal displacement of the main bridge exhibits spatial symmetry and a linear distribution along the bridge’s longitudinal axis. It shows high consistency with temperature variation trends and displays distinct seasonal variation characteristics. This pattern aligns with the deformation characteristics of the bridge, verifying the feasibility of PS-InSAR technology for measuring bridge deformation.
(2) By removing the trend component, a smoother residual sequence curve of wind-induced vibration for the bridge is obtained. This provides longitudinal and vertical displacement time-history curves of wind-induced vibrations, offering crucial data support for wind-resistant stability design and further validating the reliability of PS-InSAR technology in measuring bridge displacements.
(3) A significant benefit of this approach is its capacity to provide precise, real-time data on deformation, which facilitates the predictive maintenance and management of bridge infrastructure. The method is scalable, making it suitable for large-scale applications and supporting the broader goal of regional monitoring of major infrastructure. However, the accuracy of detecting deformations in the north–south direction is constrained by the projection geometry of SAR data, which may result in minimal errors. Several compensating techniques, such as multi-angle SAR data fusion or the use of ground control points, can be used in the future to mitigate these inaccuracies.

In conclusion, the proposed method represents a significant advancement in the field of bridge stability monitoring, offering a robust and scalable solution for infrastructure management. Future work should include comprehensive ground-truth testing on the example bridge to further validate the PS-InSAR monitoring results and deepen our understanding of wind-induced vibrations and structural stability using this novel approach. Moreover, a detailed quantitative or sensitivity analysis of wind parameters for PS-InSAR monitoring technology will be necessary for a more thorough evaluation. Future research should also focus on addressing the current limitations to enhance the method’s effectiveness in practical applications.

## Figures and Tables

**Table 1:** Classification of the wind-induced response.

Wind-Induced Response Level	Wind Pressure (Pa)	Amplitude of Vibration (cm)	Vibration Frequency (Hz)	Wind Speed (m/s)
Level 1	<80	<2	<8	<8
Level 2	[80, 110]	[2, 4]	[8, 20]	[8, 12]
Level 3	[110, 130]	[4, 6]	[20, 40]	[12, 20]
Level 4	[130, 160]	[6, 9]	[40, 60]	[20, 28]
Level 5	>160	>9	>60	>28

**Table 2:** Wind environment parameters.

Wind Environment	Item	Value
Static wind	Wind speed	9 m/s
	Wind attack angle	2°
Dynamic wind	Mean wind speed	10 m/s
	Average wind attack angle	3°
	Gust wind speed	23 m/s
	Wind attack angle	−4°
	Gust wind interval	6 min

**Table 3:** Temporal and spatial baseline distribution for SAR images.

**Serial Number**	**Image Response Time**	**Vertical Spatial Baseline (m)**	**Temporal Baseline (d)**	**Doppler Centroid Frequency Difference (Hz)**
1	20201011	354	−2.3	−8
2	20201012	284	−3.5	−17
3	20201015	0	0	0
4	20201018	315	0.2	35
5	20201021	425	0.4	31
6	20201023	−354	0.5	29
7	20201024	485	0.7	27
8	20201026	−517	0.9	25
9	20201027	618	1.5	15
10	20201030	725	3.5	17

**Table 4:** Monitoring data of wind-induced vibration response.

Wind Environment	Random Monitoring Point	Pressure/Pa	Amplitude of Vibration/cm		Vibration Frequency/Hz	Speed/(m/s)
			Monitoring Data	Data Provided by DBMM		
Static wind environment	1	115	2.5	2.3	5.4	6.8
	2	125	3.4	3.4	6.8	10.5
	3	85	1.8	1.9	8.1	12.5
	4	75	5.6	5.2	10.5	11.7
	5	85	4.8	4.7	11.5	10.4
Dynamic wind environment	1	135	5.7	5.5	13.4	9.5
	2	145	6.1	6.3	15.8	13.5
	3	118	3.5	3.5	16.7	12.4
	4	125	2.7	2.5	23.5	18.4
	5	108	1.7	1.9	35.7	14.5

## Data Availability

All research data are available from the corresponding author upon reasonable request.
